# A Comprehensive Review of the Gut–Microbiota–Brain Axis in Alzheimer’s Disease: From Pathophysiology to Potential Therapies

**DOI:** 10.3390/pathogens15070659

**Published:** 2026-06-23

**Authors:** Mairi Ziaka

**Affiliations:** Department of Emergency Medicine, Inselspital, University Hospital, University of Bern, 3010 Bern, Switzerland; mairi.ziaka@gmail.com

**Keywords:** Alzheimer’s disease, fecal microbiota transplantation, gut–microbiota–brain axis, inflammation, mild cognitive impairment, prebiotics, probiotics

## Abstract

The gut–microbiota–brain axis (GMBA), an intricate network connecting the gastrointestinal (GI) tract and the brain, plays a pivotal role in maintaining overall health and influencing disease processes. The human gut microbiota, comprising over 3000 bacterial species, regulates immune responses, hormonal signals, and metabolite production, maintaining homeostasis under normal conditions. Dysbiosis, or microbial imbalance, has been linked to various central nervous system (CNS) disorders, including Alzheimer’s disease (AD), Parkinson’s disease (PD), multiple sclerosis (MS), and autism spectrum disorder (ASD). Given the growing interest in this topic and the limited effectiveness of current therapeutic strategies for managing patients with AD, the purpose of the current narrative review is to analyze the pathophysiological role of the GMBA in the pathogenesis of AD and assess potential therapeutic strategies targeting the GMBA, particularly the microbiome and its metabolites. A comprehensive literature search was conducted using PubMed, Scopus, and Web of Science to identify clinical studies, experimental research, and review articles examining the GMBA in health and AD, as well as related therapeutic strategies. The search terms included “Alzheimer’s disease”, “neuroinflammation”, “amyloid-beta”, “tau”, “gut–brain axis”, “microbiome”, “short-chain fatty acids”, “probiotics”, “prebiotics”, and “fecal microbiota transplantation”. In AD, altered gut microbiota composition is associated with neuroinflammation, neurodegeneration, and exacerbation of disease progression. Probiotics have shown potential in enhancing cognitive function and reducing neuroinflammation by modulating microbiota composition and influencing brain-derived neurotrophic factor (BDNF) levels. Prebiotics, through their impact on gut microbiota and metabolite production, also offer therapeutic promise by improving cognitive function and mitigating neuroinflammation. With its historical and modern applications, fecal microbiota transplantation (FMT) may represent a potential strategy for addressing dysbiosis and its neurological implications. This manuscript focuses on GMBA and its effects on neuroinflammation, neurodegeneration, and CNS health while emphasizing the need for further research into microbiome-based therapies and the gut–brain relationship in patients with AD.

## 1. Introduction

Alzheimer’s disease (AD) is a neurodegenerative disorder of the central nervous system (CNS) characterized by progressive cognitive decline, with its diagnosis primarily based on the accumulation of extracellular amyloid-beta (Aβ) plaques and intracellular neurofibrillary tangles (NFTs) formed by hyperphosphorylated tau proteins [1,2]. The accumulation of these deposits triggers neuroinflammation through activation of immune pathways, leading to synapse loss and neuronal death [3]. Furthermore, disruptions to central immune homeostasis caused by infections or traumatic brain injuries can further accelerate the progression of the disease [4]. On the other hand, accumulating evidence indicates that the gut microbiota plays a crucial role in this process. Recent studies have demonstrated an association between AD and gut microbiota dysbiosis, with gut microbes influencing brain function through inflammatory, metabolic, and neuroimmune pathways. This connection, termed gut–microbiota–brain axis (GMBA), highlights the role of inflammation in the development and progression of AD [5,6,7,8].

The intestinal microbiota, a complex ecosystem mainly consisting of bacteria but also comprising viruses, fungi, protozoa, and archaea, plays a crucial role in maintaining health by regulating host immunity and supporting intestinal barrier functions [9,10,11]. Recently, the gut microbiota has been identified as a crucial element in both sustaining CNS homeostasis and contributing to its dysfunction [12,13]. The reciprocal communication between the intestine and the brain is complex and involves various pathways, including the vagus nerve, immune system interactions, endocrine signaling, and microbial metabolites [14,15,16], including short-chain fatty acids (SCFAs), proteins, and tryptophan metabolites [17].

Experimental research has shown that AD mice exhibit more pronounced age-related microbiome changes, including increased *Bacteroides* colonization linked to amyloid accumulation. At the same time, sodium butyrate, a SCFA, has been found to alleviate AD pathology in mouse models [18]. Moreover, differences in gut microbiota between AD models and wild-type mice are highlighted, notably a decrease in SCFA-producing bacteria [19,20]. In addition, fecal microbiota transplantation (FMT) from wild-type to AD mice has led to reduced amyloid deposits, supporting a contributory role of gut microbes in AD pathogenesis [19,21]. Consistent with these findings, human studies have also shown that patients with AD or mild cognitive impairment (MCI) exhibit alterations in microbiota composition compared to controls, including reduced diversity of SCFA-producing species [22,23,24,25,26]. However, clinical studies in patients with AD and MCI have reported inconsistent findings and are subject to several methodological limitations, highlighting the need for cautious interpretation of the available evidence.

Although the mechanisms underlying the gut–microbiota–brain interaction are not yet fully understood [27], the limited effectiveness of current therapeutic options for managing AD suggests that targeting the microbiota and its metabolites could offer new diagnostic and therapeutic opportunities. This work aims to comprehensively explore the GMBA in AD and assess potential therapeutic strategies. It evaluates the benefits of interventions such as probiotics, prebiotics, and FMT in modulating this axis and their impact on disease management.

## 2. Search Strategy

A comprehensive literature search was conducted using PubMed, Scopus, and Web of Science to identify studies focusing on the GMBA in health and AD, as well as associated therapeutic strategies. Search terms included “Alzheimer’s Disease”, “neuroinflammation”, “amyloid-beta”, “tau”, “gut-brain axis”, “microbiome”, “short chain fatty acids”, “probiotics”, “prebiotics”, “fecal microbiota transplantation” and “therapeutic targets” either individually or in combination. Boolean operators (AND, OR) and truncation techniques were applied to improve the precision and relevance of the search results. The search was restricted to articles published in English from 2014 to 2026, although key seminal studies published before this period were also included to provide historical context and foundational insights. The final literature search was performed on 7 June 2026.

Titles and abstracts were initially screened for relevance, followed by a full-text review of eligible studies. The final selection included primary research studies, alongside systematic reviews, meta-analyses, and narrative reviews to provide historical context, highlight key themes and trends, and identify seminal works. Narrative reviews, authored by experts, offer critical analysis, synthesized interpretations, and expert perspectives, helping to identify research gaps and controversies that inform the research questions addressed in this review. This narrative review qualitatively synthesizes and thematically organizes preclinical, translational, and clinical studies on the GMBA in AD, without application of a formal systematic synthesis framework.

The inclusion criteria comprised:

1. Peer-reviewed studies investigating the interplay between the gut microbiome and AD, particularly its influence on inflammation, Aβ, and tau pathologies.
2. Research exploring therapeutic interventions targeting the GMBA, such as probiotics, prebiotics, or FMT.
3. Clinical, preclinical, or translational studies relevant to AD and GMBA.
4. Randomized controlled trials (RCTs), cohort studies, case-control studies, and animal models with translational value.

Studies were excluded if they:

1. Primarily focused on unrelated neurodegenerative diseases, such as Parkinson’s disease (PD) or Huntington’s disease.
2. Lacked direct relevance to the GMBA in AD pathophysiology or treatment.
3. Were conference abstracts or non-peer-reviewed publications.

## 3. The Gut–Brain Axis in Health and Disease

### 3.1. Gut–Microbiota–Brain Axis

**Figure 1:**
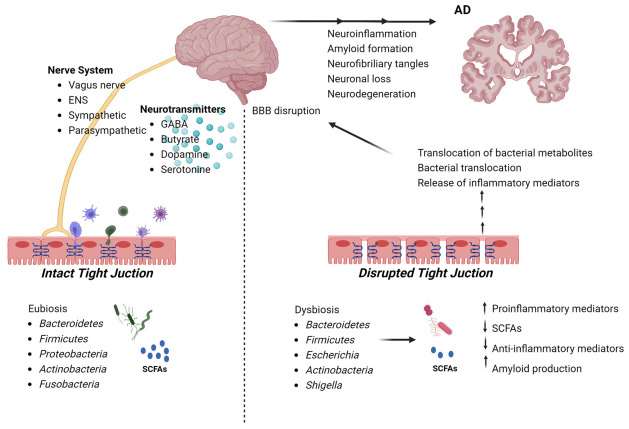
The brain communicates with the gut microbiota through intricate pathways involving neuronal pathways, the immune system, and microbial metabolites. When gut dysbiosis arises, it triggers immune activation, disturbs neurotransmitter levels, and interferes with vagus nerve signaling. Disruption of the gastrointestinal barrier permits bacterial translocation and initiates inflammatory processes, which in turn lead to the release of pro-inflammatory cytokines that increase the permeability of the BBB. As a result, harmful microbial byproducts may reach the brain or indirectly influence it, adversely affecting neurological function. Dysbiosis reduces the availability of beneficial metabolites like SCFAs while enhancing the production of harmful substances such as amyloids and LPS, contributing to compromised barrier integrity and enhanced inflammation.

The human microbiota, consisting of a diverse community of commensal and symbiotic microorganisms, reaches concentrations of over 10^13^ to 10^14^ cells per gram of content in the large intestine [28,29,30]. Among the 3000 bacterial species present in the mammalian gut, the predominant phyla include *Bacteroidetes*, *Firmicutes*, *Verrucomicrobia*, *Proteobacteria*, and *Actinobacteria* [31,32,33,34]. A well-balanced gut microbiota under normal physiological conditions plays a crucial role in maintaining homeostasis by enhancing immune function, regulating hormonal signals, and producing a wide array of metabolites, whereas in pathological states, shifts in the relative abundance of bacterial species disrupt this balance, resulting in a condition described as “dysbiosis” [35,36]. In recent years, accumulating evidence has highlighted the intricate link between gut and brain health, revealing several potential mechanisms (Figure 1); gastrointestinal (GI) dysfunction and symptoms are increasingly associated with various CNS disorders, with some conditions, such as AD, PD, multiple sclerosis (MS), and autism spectrum disorder (ASD), showing GI disturbances that may precede the onset of central neurological symptoms [11,37,38].

The GMBA describes a complex network connecting the gut and brain, integrating the CNS, enteric nervous system (ENS), and gut microbiota [39]. This connection operates through neural, endocrine, and immune pathways, with the vagus nerve as the main neural link and the hypothalamic-pituitary-adrenal (HPA) axis handling stress responses through hormonal signals [40,41,42,43,44,45]. Immune components like cytokines and chemokines interact with microbial signals, while the gut microbiota produces metabolites such as SCFAs, neurotransmitters, and neuromodulators that impact brain function and behavior [46,47,48,49,50,51,52,53]. Moreover, recent findings suggest that alterations in gut microbiota can influence neuroinflammation, affecting microglial activity and overall CNS health (Figure 1) [54,55].

### 3.2. Gut–Microbiota–Brain Axis in Alzheimer’s Disease

The composition of gut bacteria significantly influences age-related neurological disorders, including mood disorders and AD. Various factors affect gut microbiota, such as internal components like genetic variations, immunity, and metabolites, as well as external influences such as diet, lifestyle, and inflammatory insults. The gut microbiota produces crucial signaling molecules, including choline, tryptophan, and SCFAs, and regulates the secretion of hormones like leptin and ghrelin, which play essential roles in regulating CNS functions [56,57]. Aging significantly alters gut microbiota, increasing pro-inflammatory bacteria like *Bacillus fragilis* and *Faecalibacterium prausnitzii* while decreasing anti-inflammatory species such as *Eubacterium rectale* (*E. rectale*), *Lactobacillus*, *Bifidobacterium*, and *Ruminococcus*. This imbalance leads to local inflammation, greater GI permeability, and compromised blood-brain barrier (BBB) function, ultimately fostering neuroinflammation (Figure 1) [58,59]. Particularly, in patients with AD, Cattaneo et al. [60] identified a higher abundance of pro-inflammatory bacteria, such as *Escherichia/Shigella*, and a lower abundance of anti-inflammatory bacteria, like *E. rectale*, compared to healthy controls, using quantitative polymerase chain reaction (PCR) to analyze stool samples from cognitively impaired older adults with amyloidosis [60]. The findings from Vogt et al. [25] corroborate these results, as they employed 16S ribosomal ribonucleic acid (rRNA) gene sequencing to examine and categorize the bacterial profiles in fecal samples from both AD patients and healthy controls. Their research revealed that individuals with AD had a markedly reduced diversity in their gut microbiota compared to those without the disease. Specifically, they observed a decrease in *Firmicutes* and an increase in *Bacteroidetes* at the phylum level among AD patients [25]. Finally, the findings from the research above are further reinforced by experimental studies, which demonstrate that animal models have consistently shown a link between altered gut microbiota and AD manifestations [61,62].

Given the complexity of AD pathophysiology and the unclear mechanisms underlying both its generation and progression, experimental research has employed methods such as antibiotic treatments, germ-free or gnotobiotic models, and FMT. These studies have demonstrated that administering antibiotics or maintaining amyloidosis model mice (APP/PS1) in germ-free conditions can reduce cerebral amyloid plaque deposition [63,64]. However, although studies in animal models indicate that microbiota transfer can modulate disease-related phenotypes, these findings do not establish causality in humans.

Building on these findings, another area of research explores how peripheral amyloid proteins might contribute to amyloid accumulation in the brain. Experimental studies propose that these proteins could retrogradely transport via the vagal nerve or bloodstream, leading to brain amyloid deposition [65,66,67]. This theory is supported by preclinical research showing that certain bacteria produce extracellular amyloid fibers, such as curli, which have a similar beta-sheet structure to amyloid proteins and for which robust, well-established evidence exists. Specifically, bacterial-derived amyloids, including those from *Pseudomonas fluorescens* (FapC), *Escherichia coli* (*E. coli*) (curli), *Staphylococcus aureus* (phenol-soluble modulins), *Salmonella typhimurium* (*S. typhimurium*) (CsgA), and *Bacillus subtilis* (TasA), have been linked to AD pathology through their role in promoting Aβ oligomer and fibril formation [68]. Although the amino acid sequences of human and bacterial amyloid proteins are not identical, they exhibit a conserved cross-β-sheet quaternary structure characteristic of all amyloid proteins [69]. During infections-particularly those caused by *E. coli* or *S. typhimurium*, such as sepsis, GI inflammation, and urinary tract infections-curli fibers are recognized by the immune system, leading to the release of cytokines and chemokines, as suggested by experimental and clinical evidence [70,71,72]. Moreover, curli fibers have been identified as ligands recognized by Toll-like receptor 2 (TLR2) [69]. In addition, based on growing evidence that amyloid-induced activation of the NLRP3 (NOD-, LRR- and pyrin domain-containing protein 3) inflammasome upregulates caspase-1 and leads to the production of interleukin (IL)-1β [73,74,75,76], recent experimental research has used curli fibers produced by *Salmonella enterica* serovar Typhimurium and *E. coli* to study bacterial amyloid-induced activation of the NLRP3 inflammasome. The authors reported that stimulation by curli fibers results in caspase-1–mediated IL-1β production and that TLR2 is a major contributor to this pathophysiological process [70].

Current understanding points to neuroinflammation as a crucial factor linking gut microbiota alterations to AD progression. Aberrant activation of glial cells in AD is thought to disrupt brain homeostasis and may contribute to disease worsening. These hyperactive glial cells are associated with increased Aβ toxicity and accumulation and are suggested to promote the release of pro-inflammatory cytokines and reactive oxygen species. These inflammatory cascades may damage neurons, aggravate tau pathology, and accelerate disease progression [77,78,79,80,81,82]. Essential for Aβ clearance, microglia frequently form clusters around amyloid deposits, which helps mitigate neurotoxic damage and limits the addition of new Aβ to existing plaques [83,84,85,86,87]. Importantly, microglia’s proper maturation and functioning rely on the gut microbiota under normal conditions, emphasizing its crucial role in maintaining neuroinflammatory balance [88]. Indeed, research indicates that decreased gut microbiota complexity, resulting from germ-free conditions or antibiotic treatments, negatively impacts microglial development and function. Both germ-free and specific pathogen-free mice with compromised gut microbiota display immature microglial characteristics and altered cell proportions. This developmental impairment can be alleviated by supplementing with SCFAs or reintroducing a diverse microbiota, highlighting the crucial role of microbial metabolites in maintaining microglial health [88].

**Figure 2:**
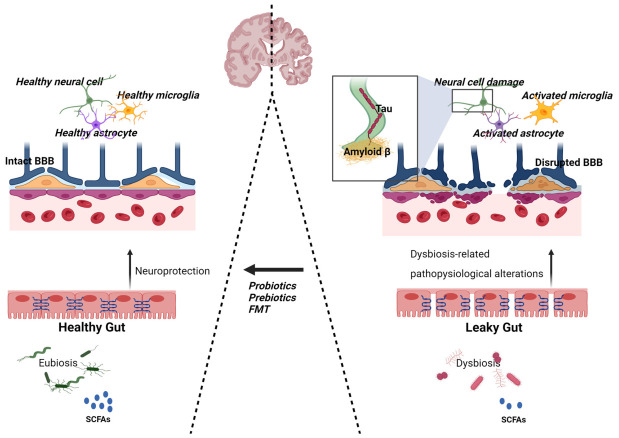
Dysbiosis plays a significant role in driving neuroinflammation, neuronal cell death, and the disruption of the BBB. This process is marked by the activation of astrocytes and microglia, which further exacerbate neuronal damage. Key contributors to AD pathology include amyloid plaque formation, tau hyperphosphorylation leading to NFT pathology, persistent neuroinflammation, oxidative stress, and the translocation of gut-derived metabolites, inflammatory mediators, and bacterial compounds through a compromised intestinal and BBB. These factors collectively lead to synaptic dysfunction and neuronal loss. Therapeutic approaches aimed at restoring gut microbiota balance-such as FMT, prebiotics, and probiotics-hold promise in promoting neuroprotection. These interventions can help to restore gut barrier integrity, decrease peripheral inflammation, mitigate BBB disruption, regulate brain immune cell activity, and enhance cognitive function, ultimately slowing the progression of AD. By reconditioning the gut–brain axis, these strategies offer a potential pathway to mitigate the complex interplay of amyloid plaque formation, tau pathology, and neuroinflammatory processes central to AD pathophysiology.

Changes in gut microbiota can also affect the permeability of both the intestinal and the BBB. Increased permeability facilitates the entry of gut-derived molecules, such as lipopolysaccharides (LPS) and SCFAs, into systemic circulation and potentially the brain. This breach can lead to a shift from a balanced state to a pro-inflammatory environment, potentially contributing to the development of neurodegenerative diseases such as AD (Figure 2) [89]. The gut microbiota composition critically influences the gut mucosa’s protective function against pathogens. In AD, the balance of gut bacteria is disrupted, leading to a reduction in strains that normally support intestinal barrier integrity, such as *Bifidobacterium infantis* and *Akkermansia muciniphila*. Conversely, bacteria that undermine epithelial cell integrity, such as *E. coli*, Shigella, and *Helicobacter pylori* (*H. pylori*), become more prevalent in these individuals [90,91]. In addition, in AD, the reduction in butyrate-producing bacteria contributes to T-cell imbalance, increased epithelial barrier permeability, and elevated bacterial translocation [3,92,93,94,95]. This disruption leads to elevated levels of circulating LPS from Gram-negative bacteria, known as metabolic endotoxemia, which activates systemic inflammation through TLR4 signaling and contributes to BBB disruption, thereby exacerbating neuroinflammation [96,97]. Finally, common comorbidities observed in patients with AD, such as type 2 diabetes mellitus, obesity, and GI disorders including *H. pylori* infection, inflammatory bowel disease, and periodontitis, as well as commonly used medications such as proton pump inhibitors, also contribute to microbiome alterations associated with impaired intestinal permeability, BBB disruption, and neuroinflammation, indicating that microbiome-related therapeutic interventions may influence the progression of both conditions [98,99].

### 3.3. The Vagus Nerve

The vagus nerve arises from the medulla oblongata and is of mixed composition, consisting of approximately 80% sensory (afferent) fibers and 20% motor (efferent) fibers, including parasympathetic fibers [100,101,102,103]. It mediates communication between the CNS and peripheral organs, including the lungs, heart, GI tract, and immune system. It regulates a wide range of physiological processes, including heart rate, cardiac contractility, and blood pressure regulation [104]; bronchoconstriction [105]; intestinal peristalsis, gastric secretion, and pancreatic function [106]; immune responses and neuro-immune interactions [107]; and mood and stress responses [108,109,110]. In addition, vagal afferents, which are considered multimodal, are capable of detecting diverse stimuli, including mechanical (e.g., tension and stretch), chemical (e.g., neurotransmitters and bacterial metabolites such as SCFAs, indoles, bile acids, and LPS via cytokine signaling), and hormonal signals [55,111,112,113,114,115,116].

Accumulating evidence highlights the anti-inflammatory capacities of the vagus nerve, which are mediated through several physiological pathways [107]. First, vagal afferent fiber–mediated activation of the HPA axis leads to the secretion of adrenocorticotropic hormone and the subsequent production of cortisol by the adrenal glands, thereby mitigating immune responses and exerting an anti-inflammatory effect [117,118,119]. Second, the cholinergic anti-inflammatory pathway involves vagal efferent fibers that synapse onto enteric neurons, leading to the release of acetylcholine at synaptic junctions. Acetylcholine then binds to α7-nicotinic acetylcholine receptors (α7nAChR) on local macrophages, inhibiting the release of tumor necrosis factor (TNF)-α [108,119]. Moreover, it has been convincingly demonstrated in recent years that the cholinergic anti-inflammatory pathway, through activation of α7nAChR, contributes to the suppression of inflammatory and immune-mediated pathological processes [120,121] by inhibiting nuclear factor kappa-B (NF-κB) signaling pathways [122] and activating other immunomodulatory pathways, such as Jak/STAT3 (janus kinases/signal transducer and activator of transcription 3) [123,124,125] triggering the secretion of anti-inflammatory mediators including IL-10 [126]. Lastly, the splenic sympathetic anti-inflammatory pathway contributes to this effect. In this pathway, the vagus nerve stimulates the splenic sympathetic nerve, resulting in the release of noradrenaline at the distal end of the splenic nerve [127,128,129]. Noradrenaline binds to β2-adrenergic receptors on splenic lymphocytes, which then release acetylcholine. Similar to the second pathway, this mechanism mitigates TNF-α release from splenic macrophages via α7nAChR [119,129]. Furthermore, the trafficking of vagally modulated intestinal immune cells from the gut to the spleen may influence splenic immune responses [130]. Moreover, catecholamine-mediated activation of vagal afferent fibers modulates sympathoadrenal activity by providing a negative feedback mechanism [131,132]. Circulating epinephrine, and to a lesser extent norepinephrine, from the adrenal glands enhances adrenergic signaling on splenic immune cells and blood vessels, supporting the systemic stress response [131,133].

Acetylcholine and forebrain cholinergic neurons play a significant role in brain functions such as learning, memory, and cognition [134,135]. Moreover, acetylcholine has been shown to regulate consolidation, reconsolidation, extinction, encoding, memory recall, and acquisition [136]. Basal forebrain cholinergic neurons originate in the basal forebrain and project to brain regions such as the hippocampus and cortex, which are involved in processes like memory and learning [137]. It is well established through experimental research that damage to basal forebrain cholinergic neurons that innervate the cortex can lead to cognitive dysfunction and attention deficits [138,139]. Additionally, as highlighted by clinical and experimental research, patients with AD exhibit severe neurodegeneration, loss of cholinergic neurons, a marked insufficiency of acetylcholine, and impaired choline acetyltransferase activity [140,141,142]. This is further supported by evidence showing that potentiation of cholinergic transmission in individuals receiving cholinesterase inhibitors is associated with improvements in attention [2]. Thus, therapies that enhance acetylcholine levels and activity, such as cholinergic treatments combined with other interventions, may represent effective strategies for improving symptomatic outcomes and functional performance [143].

Activation of vagus nerve afferent fibers also leads to the release of catecholamines from the locus coeruleus, which is one of the earliest sites affected by tau, into multiple brain regions, including areas involved in cognitive functions such as memory, as well as regions severely affected in AD, such as the hippocampus [144,145,146,147]. The released noradrenaline exerts its effects on neuroglia and astrocytes by exhibiting anti-inflammatory and neurotrophic properties [147]. It also regulates synaptic plasticity and function, with pleiotropic effects that vary according to brain region and cellular subpopulations. Furthermore, stimulation of the locus coeruleus promotes dopamine release in the hippocampus, influencing neuronal plasticity and excitability, and contributing to the consolidation of routine or “everyday” memory [144,148,149].

Growing evidence highlights the vagus nerve as a central player in the gut–brain axis, contributing significantly to the pathophysiology of various disorders, including neurodegenerative diseases such as AD [109,110]. Indeed, experimental studies highlight the interaction between the gut microbiome and the vagus nerve, which conveys signals from the GI tract to the nucleus tractus solitarius and then to the central autonomic network, including the hypothalamus, thalamus, amygdala, insula, and prefrontal cortex, thereby influencing emotion and motivation [150,151].

As early as the 2000s, it was demonstrated that alterations in the gut microbiome can induce emotional and behavioral changes through increased c-Fos expression in neurons of the vagal ganglia, for example, following oral inoculation with *Campylobacter jejuni* [152]. Experimental research in male mice receiving *Lactobacillus rhamnosus* (*L. rhamnosus*) following sham surgery demonstrated a reduction in anxiety-like behavior and suppression of the HPA axis. These effects were associated with an increase in splenic T regulatory (Treg) cells and a decrease in activated hippocampal microglia. Interestingly, vagotomy eliminated the anxiolytic effects and the suppression of the HPA axis, and resulted in a significant increase in activated microglia in the hippocampus [153]. Lee et al. [154] investigated the effects of FMT from geriatric humans and aged mice into young mice and observed significant cognitive decline compared with transplants derived from young adults and young mice. *Paenalcaligenes hominis* and *E. coli* were associated with significant cognitive impairment and colitis in specific pathogen–free mice. In contrast, celiac vagotomy significantly alleviated the development of cognitive deficits in mice exposed to *Paenalcaligenes hominis*, but not in those exposed to *E. coli* [154]. Bravo et al. [155] demonstrated that chronic administration of *L. rhamnosus* to healthy male BALB/c mice resulted in increased gamma-aminobutyric acid (GABA) receptor expression in cortical regions, including the cingulate and prelimbic cortex, and decreased expression in the hippocampus, amygdala, and locus coeruleus. Moreover, *L. rhamnosus*-treated mice exhibited reduced stress-induced corticosterone levels as well as decreased anxiety- and depression-like behaviors. These effects were not observed in mice that had undergone vagotomy [155]. Moreover, activation of the vagus nerve by non-pathogenic bacteria such as *Lactobacillus lactis* results in increased activity of the sympathetic nervous system, effects that are eliminated by subdiaphragmatic vagotomy [156,157]. Additionally, cell-specific transneuronal tracing has shown that signals from the right vagal afferent ganglion are transmitted via glutamatergic neurons of the dorsolateral parabrachial nucleus, potentially reaching the substantia nigra, where activation of this neural circuit induces dopamine release and reward-related behaviors [150].

BBB dysfunction contributes significantly to the pathophysiology of a variety of CNS inflammatory diseases, including AD, by promoting pathological vascular leakage of pathogenic molecules such as pro-inflammatory mediators and reactive oxygen species, and by permitting the infiltration of inflammatory cells into the CNS [158]. It has been suggested that the vagus nerve plays a major role in transmitting signals from the gut microbiota to the choroid plexus, thereby maintaining the integrity of the BBB [159]. This is consistent with multiple studies showing that vagus nerve stimulation can restore BBB integrity by suppressing inflammatory responses and by limiting the upregulation of the transcellular transport pathway [160,161,162]. Furthermore, vagus nerve stimulation activates α7nAChRs, thereby inhibiting the activity of pro-inflammatory cells and the production of pro-inflammatory mediators, primarily protecting the BBB from their deleterious effects [163].

The vagus nerve is implicated in intestinal-to-cerebral seeding and spreading of neurodegenerative molecules, such as Aβ and misfolded α-synuclein, as well as in the cellular mechanisms and receptor pathways that contribute to the development of a “bottom-up” signaling axis [42,66,164,165,166]. Within this signaling cascade, sensitive receptors on vagal afferent terminals detect changes in the intestinal microenvironment and mediate the transmission of signals associated with inflammatory responses, pathological protein species, and metabolic alterations to the brain [42]. Gut microbiota alterations, intestinal barrier dysfunction, and activation of intestinal immune mechanisms are associated with vagal nerve dysfunction, leading to neurochemical and behavioral consequences [155,167]. Indeed, experimental evidence highlights that chronic treatment with *Lactobacillus* strains modulates mRNA expression of GABA receptors in the hippocampus, an effect that was absent in vagotomized control animals [155]. In addition, dysbiosis is associated with cognitive dysfunction through the local, direct, or indirect production of pathogenic proteins such as LPS and amyloid proteins, which can trigger neurodegenerative mechanisms, particularly via intestinal and BBB dysfunction. Indeed, the intestinal accumulation of harmful α-synuclein is directly influenced by bacterial amyloid in a “cross-seeding” manner, subsequently entering the brain via the vagus nerve and contributing to neurodegenerative cascades [168]. Furthermore, given the prion-like properties of Aβ, it has been suggested that extracranial Aβ, specifically Aβ of intestinal origin, may contribute to the cerebral Aβ load. Experimental research in ICR mice, in which Aβ was injected into the GI tract, revealed the presence of Aβ in the vagus nerve and brain after one year, which was associated with intestinal dysfunction and cognitive decline [66]. In a similar manner, Aβ and tau fibril formation, mediated by intestinal and cerebral activation of the CCAAT/EBPβ/AEP pathway and followed by transmission to the brain via the vagus nerve, may also contribute to cognitive dysfunction in patients with AD [165].

Targeted stimulation of specific neural networks has been investigated as a potential neuromodulatory strategy to improve cognition and functional impairment in patients with neurodegenerative diseases [169,170]. Experimental and clinical studies demonstrate the beneficial effects of modulating the gut–brain axis, such as through vagus nerve stimulation (VNS), in neuropsychiatric disorders and conditions involving inflammatory processes [108,171,172,173], including AD [2]. Available research highlights that vagal activity is involved in the pathophysiology of cognitive disturbances, such as memory decline, and that VNS can enhance these functions by promoting neural plasticity in brain regions associated with memory consolidation, such as the hippocampus [174,175,176,177]. A systematic review analyzing the effects of VNS in experimental and clinical studies, while noting that VNS is currently approved by the Food and Drug Administration for refractory epilepsy, depression, migraine, and post-ischemic stroke rehabilitation, reported that VNS resulted in improved cognition as well as enhanced mobility and balance. These effects were attributed to multiple mechanisms, including increased dopaminergic neuron activity, upregulation of α7nAChR expression, attenuation of neuroinflammation, reduced apoptotic mediators, and modulation of microglial and astrocytic populations [178]. Moreover, Yuan et al. [179] demonstrated, in an experimental model of abdominal surgery–induced intestinal ileus, that abdominal surgery upregulates M1 macrophages and elevates pro-inflammatory cytokines, processes that could be suppressed with central vagal activation, highlighting its implication in modulating dysbiosis and intestinal function [179].

## 4. Potential Gut Microbiome-Targeted Therapies in the Management of Alzheimer’s Disease

### 4.1. Probiotics

Classified as “live organisms that confer health benefits to the host when administered in adequate doses”, probiotics, which include species such as *Lactobacilli*, *Bacillus* species, various strains of *Bifidobacteria*, *Streptococcus thermophilus*, *E. coli* strain Nissle 1917, and yeasts like *Saccharomyces boulardii* and *Saccharomyces cerevisiae* [180], offer numerous health benefits by balancing the body’s pH levels, protecting the gut lining, promoting competitive exclusion, producing antimicrobial metabolites, modulating immune responses, and enhancing brain-derived neurotrophic factor (BDNF), which is crucial for brain health (Figure 2) [181]. BDNF represents a well-studied growth factor in the mammalian brain, very important for supporting nerve growth and maturation during developmental stages, while also playing a significant role in regulating synaptic transmission and plasticity in adulthood [182,183]. Patients with AD show markedly reduced levels of serum BDNF when compared to healthy individuals [184], whereas the reduction in BDNF levels is associated with several detrimental processes, including tau protein phosphorylation, the buildup of Aβ, increased neuroinflammation, and the onset of neuronal apoptosis [185]. The interplay between inflammation and neuroplasticity, mediated by BDNF, regulates neurotransmitter release, such as glutamate and GABA, through the activation of NF-κB [186,187]. Evidence suggests that combining *Lactobacillus* and *Bifidobacterium* can effectively raise BDNF levels in individuals suffering from neurological conditions [184]. Moreover, a double-blind clinical trial showed that AD patients who received a probiotic mixture of *Lactobacillus* and *Bifidobacterium* experienced significant improvements in cognitive function compared to those given a placebo [188]. Beyond their effects on BDNF, probiotics can directly influence CNS biochemistry by altering levels of GABA, serotonin (5-hydroxytryptamine; 5-HT), and dopamine (DA), which may in turn affect mood and behavior [155,189,190].

Furthermore, probiotics can provide health benefits when given in sufficient quantities, potentially through their anti-inflammatory or antioxidant effects, with evidence indicating that they may also influence the CNS and behavior by modulating the GMBA, thereby addressing neuroinflammation [16,191,192,193]. Experimental studies using mouse models have highlighted that the *Lactobacillus casei* strain *Shirota* can effectively reduce neuroinflammation, showing potential benefits in reducing AD [194]. Leblhuber et al. [195] observed that probiotic administration increased serum kynurenine, likely due to the activation of immune cells, which could help remove amyloid aggregates and damaged cells but might also impair gut barrier function and aggravate neurodegenerative conditions [195]. Indeed, kynurenine, a marker of immune system activation linked to tryptophan metabolism through indoleamine 2,3-dioxygenase-1, serves as a protective mechanism against inflammation by stimulating Tregs that reduce inflammation and provide antiproliferative and immunosuppressive effects [196,197,198]. Furthermore, early-stage AD 3xTg-AD mice treated with the SLAB51 probiotic formulation showed changes in gut microbiota and its metabolites, which in turn affected plasma levels of inflammatory cytokines and key metabolic hormones targeted in neurodegeneration. This treatment led to partial restoration of two impaired neuronal proteolytic pathways-the ubiquitin-proteasome system and autophagy-and resulted in reduced cognitive decline, decreased brain damage, and lower accumulation of Aβ aggregates compared to control mice [199]. In a recent study, AD rats treated with probiotics (*Lactobacillus reuteri*, *L. rhamnosus*, and *Bifidobacterium infantis*) showed significant improvements in spatial memory, reduced Aβ plaques, decreased oxidative stress, and lower inflammation markers IL-1β and TNF-α compared to controls [200]. Studies show that probiotics with *Bifidobacterium breve* and *Bifidobacterium infantis* decrease Aβ deposition, IL-1β, and TNF-α while increasing superoxide dismutase [63] levels in the hippocampus of Aβ-induced AD mice. Some strains of Bifidobacterium also improve cognitive function and reduce immune responses and inflammation by raising plasma acetate levels, demonstrating their ability to suppress Aβ-induced toxicity and normalize gene expression, particularly BDNF, which promotes neuronal survival in AD [200,201,202]. Bonfili et al. [199] conducted a preclinical study on AD mice and discovered that the lactic acid bacteria and bifidobacteria (SLAB51) probiotic blend, which includes *Streptococcus thermophilus*, *Lactobacilli*, and *Bifidobacteria*, was effective in reducing Aβ burden, alleviating cortical atrophy, and restoring the ubiquitin proteolytic system and autophagy [199]. Furthermore, *Lactobacillus plantarum* C29 was found to regulate microglia activation and reduce Aβ deposition in 5xFAD transgenic mice [203].

Age-related diseases, including AD, are influenced by genetic factors and stress management, with the combination of genetic vulnerabilities and environmental stressors contributing to inflammatory conditions like AD [204]. Thevaranjan et al. [205] found that microbial dysbiosis associated with aging can lead to increased gut permeability and inflammation. As mentioned above, with advancing age, the gut microbiota undergoes significant changes, including an increase in *Proteobacteria*, a decline in beneficial bacteria such as *Bifidobacteria*, and a decrease in neuroprotective SCFAs [206]. Indeed, these findings are in accordance with the findings of a meta-analysis suggesting that probiotics, due to their anti-inflammatory and antioxidant properties, may improve cognitive function in patients with AD and MCI [207]. In a randomized, double-blind, placebo-controlled multicenter trial, probiotics were shown to modify the gut microbiome, enhance mental flexibility, and reduce stress in healthy older adults. At the same time, another study demonstrated that probiotic milk significantly improved cognitive scores, reduced oxidative stress, and lowered inflammation markers in AD patients over a 12-week period, findings which are further supported by experimental research [207,208,209]. An uncontrolled clinical study found that 90 days of probiotic-fermented milk supplementation (2 mL/kg daily) improved cognitive deficits in Alzheimer’s patients by addressing systemic inflammation, oxidative stress, and blood cell damage, suggesting kefir could be a promising adjunct therapy for slowing disease progression [82].

Immunoregulatory microbes are increasingly recognized for their ability to induce Tregs, crucial for maintaining immune balance and limiting harmful inflammatory processes [210,211,212]. Indeed, clinical and experimental studies have shown that certain commensal bacteria, including *Bifidobacteria infantis* and *Faecalibacterium prausnitzii*, can promote the induction of Tregs and IL-10 in the gut [213,214]. As mentioned above, dietary components such as probiotics and prebiotics have the ability to impact health by altering the composition and function of the mucosal immune system and gut microbiota. They can increase pathogen exclusion and enhance intestinal epithelial integrity through competition or inhibition of adherence [215,216,217,218,219,220]. Furthermore, these dietary factors can influence both the gut and systemic immune systems, particularly by stimulating various components of innate and adaptive immune responses. This involves the activation of Tregs and regulatory B cells (Breg), T helper cell type (Th)1, Th2, and Th17 responses, alongside the humoral response [221,222,223,224,225].

### 4.2. Prebiotics

Prebiotics, defined as “a substrate selectively utilized by host microorganisms to confer a health benefit” [226], typically include nutrients like fiber, oligosaccharides, and polyphenols. While the definition remains a topic of debate, prebiotics are generally recognized as dietary carbohydrates that are selectively fermented by gut microbiota, influencing their composition and, in turn, offering health benefits to the host [227,228]. They have been shown to enhance the growth of beneficial bacteria [229,230,231], stimulate the immune system [54], improve intestinal barrier function, and promote the production of metabolites that benefit the host [92,95,232,233]. Rodent studies increasingly highlight the neurobiological benefits of prebiotic intake, showing significant anti-inflammatory and neuroprotective effects in disease models, along with positive impacts on behavioral outcomes like anxiety, learning, and memory (Figure 2) [234].

Prebiotic supplementation has been found to induce gene expression of both BDNF and N-methyl-D-aspartate receptor (NMDAR) in the hippocampus and dentate gyrus. Similarly, in neonatal rats, B-galactooligosaccharides (GOS) treatment led to increased hippocampal BDNF and NMDAR expression compared to placebo, with these effects still observable 26 days after the treatment was discontinued [235]. An experimental study investigating the impact of chitosan oligosaccharides (COS) on cognitive deficits and their underlying molecular mechanisms revealed that COS effectively improves cognitive impairments in an Aβ1–42-induced AD model by reducing oxidative stress and neuroinflammatory reactions [236]. Similarly, fructooligosaccharides (FOS) enhanced spatial learning and memory in a D-galactose rat model of AD [237]. However, dietary supplementation with GOS did not lead to significant differences in latency or correct choices in piglets, as measured by the T-maze [238].

SCFAs and secondary bile acids produced by gut microbiota significantly affect serotonin production in enterochromaffin cells. This serotonin synthesis, in turn, influences gut motility and brain serotonergic systems. Consequently, prebiotics that increase SCFAs and bile acid production could enhance neurological function and behavior by raising serotonin levels [239,240,241]. Despite the ongoing debate regarding systemic inflammation’s effect on central 5-HT receptors, experimental research has shown that LPS-induced inflammation leads to increased cortical 5-HT2A receptor transcripts and higher hippocampal 5-HT release. Recent findings indicate that non-digestible GOS (Bimuno^®^, BGOS) administration can reverse these changes by normalizing elevated 5-HT2A receptor expression and reducing IL-1β and TNF-α levels linked to LPS-induced inflammation [242,243,244]. In addition, the association of neurotransmitter production, particularly serotonin and GABA, with *Bifidobacterium* and *Lactobacillus* genera underscores the potential of prebiotics that foster these microbes to reduce gut dysbiosis and improve intestinal neurotransmitter levels such as GABA, thereby potentially enhancing gut health and mitigating neurobehavioral disorders associated with AD [245,246,247,248,249].

### 4.3. Fecal Microbiota Transplantation

FMT refers to the procedure of transferring fecal material, which contains a diverse array of gut microbiota from a healthy donor, into the GI tract of an individual with a condition related to dysbiosis or an imbalance in their gut microbiota [220,250]. The earliest recorded use of FMT, referred to as “yellow soup” dates back to the 4th century for treating diarrhea and food poisoning, while modern scientific documentation of FMT as a treatment for pseudomembranous colitis first appeared in 1958 [251,252]. Interest in FMT surged significantly in 2013 following the publication of an RCT highlighting its remarkable effectiveness in treating recurrent *Clostridium difficile* infection (CDI) compared to conventional therapies. This trial demonstrated a success rate of nearly 90% for FMT in severe CDI cases, which not only renewed interest in the procedure but also provided substantial validation for its potential application in modern medical practice [94,253,254,255,256].

Beyond recurrent CDI, FMT has demonstrated notable effectiveness in treating other GI disorders, such as inflammatory bowel disease [257], and is also showing promise in addressing non-GI conditions, including neurological and psychiatric disorders like acute ischemic stroke [258,259], autism [260,261], and PD [262]. Given the observed dysbiosis in the gut microbiota of AD patients, with a reduction in microbial diversity compared to healthy individuals, and the beneficial effects of prebiotics, probiotics, and antibiotics on AD prognosis, there is growing interest in exploring gut microbiota regulation, including FMT, as a potentially effective new treatment strategy for AD (Figure 2) [263]. Indeed, in an experimental study, Sun and co-workers [19] examined the impact of FMT in APP/PS1 mice, a well-established AD model. Mice receiving FMT from wild-type donors exhibited notable cognitive enhancements, reduced Aβ plaque accumulation, and lower soluble Aβ40 and Aβ42 levels compared to untreated controls. Additionally, there was an increase in proteins linked to synaptic plasticity and a significant rise in the advantageous SCFA butyrate in the gut [19]. Zhan et al. [264] found that while broad-spectrum antibiotics impaired cognitive function in wild-type mice, FMT from senescence-resistant mice restored spatial learning and memory, indicating that FMT can reverse cognitive deficits induced by antibiotics [264]. Recently, Elangovan et al. [265] explored the effectiveness of FMT for AD using a well-established mouse model, finding that a short 7-day treatment notably reduced plaque accumulation and improved behavior in familial AD (5xFAD) mice. Additionally, the study highlighted that the donor’s age significantly impacts the treatment’s effectiveness [265]. Furthermore, a study using reciprocal FMT between healthy and AD mice demonstrated that transfer of fecal material from AD mice caused memory impairment and cognitive decline in healthy hosts through increased oxidative stress and local and systemic inflammatory responses. In contrast, FMT from healthy donors to AD mice improved behavior, memory function, and cognitive outcomes [266]. Recently, using an experimental model of familial AD, Jiang et al. [267] reported that the efficacy of FMT in modulating Aβ pathophysiology, including reductions in Aβ burden via inhibition of the TLR-4-inhibitor of κB kinase-β/NF-κB signaling pathway, decreased LPS levels in the colon and hippocampus, and restoration of gut dysbiosis-is both dose- and time-dependent [267].

The data on FMT for patients with AD are limited, primarily comprising case reports and a single case series study. A case report on an 82-year-old man with recurrent CDI and AD revealed initial mild cognitive impairment, with a Mini-Mental State Examination (MMSE) score of 20 and significant memory deficits. After FMT, his MMSE score improved to 26 within four months, indicating enhanced cognition and mood, and further increased to 29, along with notable improvements in memory and social interactions, six months post-FMT [268]. Similarly, FMT in a 90-year-old woman with AD dementia, who received FMT for severe CDI, was associated with improved cognitive function as assessed by the MMSE, Montreal Cognitive Assessment, and Clinical Dementia Rating, along with alterations in gut microbiota composition and significant changes in SCFAs [269]. Furthermore, a recent study involving ten patients with dementia and severe CDI explored the effects of FMT on cognitive function and gut microbiota, revealing significant cognitive improvements and shifts in gut microbiota composition from higher *Proteobacteria* to increased *Bacteroidetes*, along with changes in amino acid metabolism pathways, suggesting that FMT could be a promising approach for delaying cognitive decline in dementia by modifying gut microbiota [270]. In a single-arm clinical study including five patients with cognitive impairment (three with severe and two with mild impairment), Chen et al. [271] demonstrated improved or maintained scores on the Montreal Cognitive Assessment-B (MoCA-B), Activities of Daily Living (ADL), and the cognitive section of the AD Assessment Scale (ADAS-Cog) after FMT. In patients with severe cognitive dysfunction, the authors reported no worsening of cognitive performance [271]. Similarly, Kim et al. [272] reported significant improvements in cognitive function tests in five patients with AD three months after FMT, which were associated with alterations in gut microbiome composition, particularly an increase in *Bacteroidaceae* and a decrease in *Enterococcaceae* [272].

Despite probiotics, prebiotics, and FMT representing promising strategies in the management of AD, this review primarily emphasizes these interventions, while other GMBA-targeted strategies such as dietary patterns, synbiotics, postbiotics and microbial metabolites, lifestyle and exercise interventions, neuromodulatory approaches, and emerging microbiome-engineering technologies are not discussed in depth. Consequently, the therapeutic framework presented is not fully comprehensive, highlighting the need for future integrative, multimodal approaches.

## 5. Limitations and Area of Future Research

Current evidence should be interpreted with caution, as existing clinical studies demonstrate notable heterogeneity in therapeutic interventions, including antibiotic therapy and FMT, as well as in the definitions of key terminology such as microbiome alterations, dysbiosis, diversity, and systemic and cerebral inflammatory responses. These limitations highlight the urgent need for standardized protocols, consistent clinical outcomes, randomized studies involving larger and more homogeneous patient populations, and thorough safety assessments and long-term outcomes [220,273]. Moreover, although gut microbiome–modifying therapies represent promising therapeutic options for patients with AD, many of the available studies have small sample sizes, which may result in random error, short durations, and variable outcomes. Long-term outcomes are also often lacking, thereby limiting the ability to draw clear conclusions regarding the association between microbiome alterations and prognosis. In addition, the effectiveness of these interventions should be evaluated across different stages of AD, as their efficacy appears to be limited in advanced stages of the disease when neurodegeneration is irreversible [220,274,275]. Indeed, as AD represents a continuum that begins prior to symptomatic manifestation, studies of the GMBA should focus on different disease stages. Human studies highlight that patients with MCI, a predementia stage, exhibit less pronounced microbiome alterations than patients with established AD. Specifically, in MCI, a decrease in *Bacteroides* and *Bacteroidetes* and an increase in *Phascolarctobacterium* are observed; additionally, a progressive intestinal enrichment of *Gammaproteobacteria*, *Enterobacteriales*, and *Enterobacteriaceae* is also observed as the disease progresses from MCI to AD, which may influence the therapeutic efficacy of microbiome-modifying interventions [23,276]. Furthermore, it should be taken into account that studies with negative or statistically non-significant results may remain unpublished, leading to publication bias and an overestimation of the therapeutic potential of gut microbiome–regulating therapies [220,274]. Additionally, despite the generally favorable safety profile of gut microbiome–regulating therapies, particularly in healthy individuals, rare but severe adverse effects, such as systemic infections, may occur in older patients [275,276]. Nonetheless, available research on the long-term effects of FMT remains limited, particularly with respect to the persistence and behavior of specific bacterial species [277,278,279]. Moreover, the longevity of the transplanted microbiome and its immune interactions with the host remain matters of concern, especially given that host gut microbiome alterations may occur long after FMT and that the long-term consequences, such as the potential development of chronic diseases and the transfer of antibiotic resistance genes, are not yet fully defined [220,280,281,282,283]. Future research should focus on addressing these issues by designing large, randomized, long-term trials and by using standardized strains, dosages, treatment durations, and well-defined assessment protocols. Particularly for FMT, the existing data are poorly defined regarding donor properties, as well as variations in preparation procedures, including sample processing, sample storage, dosage, routes of administration, and delivery methods [220]. Moreover, the study of advanced methodologies to modify the intestinal microbiome-such as microbial encapsulation, the use of bacteriophages, microbial enzyme modulators, and other bioengineered microbes producing beneficial metabolic products-could support the development of efficacious therapeutic interventions in the field [273]. Future research may also benefit from the integration of artificial intelligence and machine-learning approaches to accelerate advances in the gut–brain axis field. These tools could enable the integration of multi-omics data, including microbiome, metabolomics, inflammatory, neuroimaging, and clinical datasets, to identify robust microbiome-derived biomarkers, stratify responder and non-responder subgroups, and prioritize candidate microbial strains or metabolites for therapeutic development [284,285,286,287,288,289,290].

## 6. Conclusions

Exploring the role of the gut microbiota in AD has revealed significant links between intestinal microbial alterations and disease pathology. Evidence indicates that changes in gut microbiota can influence neuroinflammation, neurodegeneration, and CNS homeostasis through the GMBA. Emerging therapies such as probiotics, prebiotics, and FMT have shown potential in modulating disease outcomes. Despite these promising developments, the complex relationship between gut microbiota and brain homeostasis in AD requires further investigation. In particular, the long-term effects of microbiome modulation, including the persistence and stability of specific bacterial species, as well as safety concerns such as the potential transfer of unknown pathogens and the spread of antibiotic resistance, need to be carefully evaluated. Key parameters such as dosage, timing of administration, and host microbiome heterogeneity, as well as intestinal conditions and potential synergistic effects with existing AD treatments, also remain to be clarified. Continued research, particularly across different disease stages, is essential to validate these interactions, elucidate underlying mechanisms, and support the development of effective therapeutic strategies for this vulnerable patient population.

## Data Availability

Not applicable.
